# Integration of Transcriptome, Proteome, and Metabolome Provides Insights into How Calcium Enhances the Mechanical Strength of Herbaceous Peony Inflorescence Stems

**DOI:** 10.3390/cells8020102

**Published:** 2019-01-30

**Authors:** Daqiu Zhao, Yuhan Tang, Xing Xia, Jing Sun, Jiasong Meng, Jiali Shang, Jun Tao

**Affiliations:** 1 Jiangsu Key Laboratory of Crop Genetics and Physiology, College of Horticulture and Plant Protection, Yangzhou University, Yangzhou 225009, China; dqzhao@yzu.edu.cn (D.Z.); DX120180105@yzu.edu.cn (Y.T.); M160620@163.com (X.X.); jingsun@yzu.edu.cn (J.S.); jsmeng@yzu.edu.cn (J.M.); 2 Institute of Flowers and Trees Industry, Yangzhou University-Rugao City, Rugao 226500, China; 3 Ottawa Research and Development Centre, Science and Technology Branch, Agriculture and Agri-Food Canada, Ottawa, ON K1A 0A1, Canada; jiali.shang@agr.gc.ca

**Keywords:** inflorescence stem, mechanical strength, lignin, cut flower quality, calcium

## Abstract

Weak stem mechanical strength severely restrains cut flowers quality and stem weakness can be alleviated by calcium (Ca) treatment, but the mechanisms underlying Ca-mediated enhancement of stem mechanical strength remain largely unknown. In this study, we performed a comparative transcriptomic, proteomic, and metabolomic analysis of herbaceous peony (*Paeonia lactiflora* Pall.) inflorescence stems treated with nanometer Ca carbonate (Nano-CaCO_3_). In total, 2643 differentially expressed genes (DEGs) and 892 differentially expressed proteins (DEPs) were detected between the Control and nano-CaCO_3_ treatment. Among the 892 DEPs, 152 were coregulated at both the proteomic and transcriptomic levels, and 24 DEPs related to the secondary cell wall were involved in signal transduction, energy metabolism, carbohydrate metabolism and lignin biosynthesis, most of which were upregulated after nano-CaCO_3_ treatment during the development of inflorescence stems. Among these four pathways, numerous differentially expressed metabolites (DEMs) related to lignin biosynthesis were identified. Furthermore, structural observations revealed the thickening of the sclerenchyma cell walls, and the main wall constitutive component lignin accumulated significantly in response to nano-CaCO_3_ treatment, thereby indicating that Ca can enhance the mechanical strength of the inflorescence stems by increasing the lignin accumulation. These results provided insights into how Ca treatment enhances the mechanical strength of inflorescence stems in *P. lactiflora*.

## 1. Introduction

Herbaceous peony (*Paeonia lactiflora* Pall.) is a traditionally well-known flower in China with a cultivation history of approximately 4000 year. With the advancement of its industrialization in recent years, increasing attention has been given to the application of *P. lactiflora* cut-flowers, and it has been developed as a popular high-grade cut flower worldwide [1]. Industry standards specify that the upright growth of inflorescence stems, as affected by stem strength, is an important indicator in quality of the cut flowers. However, in production practice, it is often found that the inflorescence stems of numerous *P. lactiflora* cultivars are readily to bend or break and unable to support the weight of the flower, which can seriously reduce the quality of *P. lactiflora* cut flowers and their commercial value. In physics, mechanical strength is an important parameter that can be used for stem strength evaluation [2]. Therefore, it is desirable to identify certain techniques whereby the mechanical strength of *P. lactiflora* inflorescence stems can be enhanced.

Calcium (Ca), mainly found in plant cell walls, is an essential nutrient and plays an important role in maintaining the structure and function of the cell wall [3]. As an important signal molecule, Ca also plays a prominent role in regulating plant growth and development [4]. Accordingly, exogenous Ca treatment is typically employed to regulate gene expression, maintain cell wall integrity, and improve plant growth and development [5,6]. Exogenous Ca treatment has also shown increased stem diameter in roses (*Rosa hybrida* L.) [7], enhanced stem firmness of chrysanthemum (*Chrysanthemum morifolium* Ramat) [8]. Moreover, exogenous Ca treatment delayed stem bending in gerbera (*Gerbera jamesonii* cv. Tamara), which might be due to the coupling of pectin molecules, resulting in more cell wall stiffness [9]. More importantly, a significant increase in the mechanical strength of the inflorescence stems has been observed in *P. lactiflora* after exogenous Ca treatment, which might be induced by the changes of cell wall fractions [10]. In addition, recent reports also stated that this positive role of Ca might be related to the calcium ions (Ca^2+^) sensors [11,12]. However, the underlying molecular mechanisms of exogenous Ca treatment on enhancing the mechanical strength of *P. lactiflora* inflorescence stems remain largely unknown. Up to now, very limited genomic resources are available for *P. lactiflora*, with only a few nucleotide sequences in the GenBank. Therefore, it is of both theoretical and practical values to study the molecular genetics underlying the Ca-mediated responses using high-throughput technologies, particularly the prevalent deep-sequencing technology.

For non-model plants without documented corresponding genome sequences, RNA sequencing (RNA-seq) is a highly efficient method based on the next-generation sequencing technology, which can provide extensive coverage and unbiased representation of transcript abundance [13]. Furthermore, due to the discrepancies between protein levels and cognate gene abundance, it is important to explore gene expression patterns [14]. Thus, complementary analysis of the proteome using isobaric tags for relative and absolute quantitation (iTRAQ) technology combined with RNA-seq analysis could provide an important validation tool for the identification of key genes. Additionally, metabolomic profiling using liquid chromatography-electrospray ionization-tandem mass spectrometry (LC-MS/MS) can make a useful contribution to molecular genetics studies [15]. Therefore, application of an integrated approach combining transcriptomic, proteomic, and metabolomic analyses can be effective in exploring the mechanisms of how Ca application enhancing mechanical strength delineating mechanisms at the molecular level. Accordingly, in this study, we investigated the effects of nano-CaCO_3_ on improving of the mechanical strength of *P. lactiflora* inflorescence stems and the underlying mechanisms of this improvement. Assessments included measurement of morphological indices and photosynthetic characteristics, integration of transcriptome, proteome, and metabolome, observations of microstructures, and analysis of cell wall compositions. Collectively, our results would provide a more comprehensive understanding of the effects of Ca application on *P. lactiflora* cut flowers.

## 2. Materials and Methods

### 2.1. Plant Materials and Treatment

Experimental treatment was performed in the field of the germplasm repository of Horticulture and Plant Protection College, Yangzhou University, Jiangsu Province, China (32°23′ N, 119°24′ E). *P. lactiflora* cultivar ‘Hongyan Zhenghui’, planted into 80 rows (two plants per row), was used as the experimental material. The plants were transplanted here since 2009 and grew well in field conditions with sufficient light and water supply. After leaf expansion in March 2016, the leaves and inflorescence stems of 30 rows plants were sprayed with 2% nano-CaCO_3_ once a week until the full-bloom stage (in total of five times), whereas another 25 rows plants was treated with deionized water as the Control group. Through the course of the *P. lactiflora* development, its morphological indices and photosynthetic characteristics were measured every 8 days from 4 April to 28 April covering four developmental stages (S1, flower-bud stage; S2, pigmented stage; S3, unfold-petal stage; and S4, full-flowering stage), and the top 12 cm of thirty inflorescence stems were then collected as the samples for each stage. Subsequently, the part between top 5 cm to top 8 cm inflorescence stem was fixed in 2.5% glutaraldehyde solution for microstructure observation, and the other part was immediately frozen in liquid nitrogen, and then stored at −80 °C until further analysis.

### 2.2. Morphological Indices and Photosynthetic Characteristics Measurements

Morphological indices were measured as described previously [16]. Portable photosynthesis system (LI-6400, Li-Cor, Lincoln, NE, USA) was used to determinate photosynthetic characteristics from 8:00 to 10:00 am local time before each collection. The standard leaf chamber had dimensions of 2 cm × 3 cm, the photosynthetic photon quanta flux density (PPFD) was set at 1000 µmo1·m^−2^·s^−1^ using a self-taking red and blue LED source. Net photosynthesis rate (*Pn*) and transpiration rate (*Tr*) were also recorded in the system. All the parameters were measured on fully expanded leaves at the fourth apical node from the plant apex of ten different plants one group on the same day.

### 2.3. Transcriptome with RNA-Seq and Data Analysis

Total RNAs extracted from the inflorescence stems of the Control and Nano-CaCO_3_ treatment at S4 using a MiniBEST Plant RNA Extraction Kit (TaKaRa, Kusatsu, Shiga, Japan) were used for transcriptome sequencing. Six libraries (Control and nano-CaCO_3_ treatment, three replicates) were prepared and sequenced by Beijing Genomics Institute Co., Ltd. (Shenzhen, China) using an Illumina HiSeq™ 4000 system (Illumina Inc., San Diego, CA, USA). After raw read filtering, transcriptome de novo assembly was performed using the short reads assembling program Trinity, which combined three independent software modules [17]. The resulting sequences of Trinity were called Unigenes. Unigene annotation was performed using various bioinformatics databases, including the nonredundant protein (NR), nonredundant nucleotide (NT), Interpro and gene ontology (GO,), cluster of orthologous groups of proteins (COG), Kyoto encyclopedia of genes and genomes (KEGG,). The unigene expression level was calculated using the fragments per kilo bases per million reads (FPKM) method [18]. The threshold for significantly differentially expressed genes (DEGs) was set at a fold change ≥2.0 and a FDR value <0.001. DEG functions were explored through GO and KEGG pathway analysis and the terms which *q*-value ≤0.05 were defined as significant enriched. This was performed to identify significantly enriched metabolic pathways.

### 2.4. Proteome with ITRAQ and Data Analysis

The samples used in RNA-seq were used to perform proteome analysis. Protein extraction was performed exactly as described previously [19]. After trypsin digestion, peptides were desalted with a C18 column (Phenomenex, Torrance, CA, USA) and dried in a spin vacuum. The desalted peptides (100 µg) were labeled with iTRAQ 6-plex reagent in (Sciex, Framingham, MA, USA) 200 mM TEAB according to the manufacturer’s instructions. Labeling was as follows Control-1, 114; Control-2, 115N, Control-3, 115C; Nano-CaCO_3_-1, 116N; Nano-CaCO_3_-2, 116C; and Nano-CaCO_3_-3, 117N. The labeled peptides with different reagents were combined, vacuum-dried, and separated on a LC-20AB HPLC Pump system (Shimadzu, Kyoto, Japan) coupled with a high pH RP column. Subsequently, the peptides were subjected into the tandem mass spectrometry Q EXACTIVE (Thermo Fisher Scientific, San Jose, CA, USA) for DDA detection by nanoelectrospray ionization.

Raw data were processed with OpenMS software v2 (Free University of Berlin, Berlin, Germany) and searched against the SwissProt and common MS contaminant database using Mascot Software v2 (Matrix Science, London, UK). Trypsin was chosen as the enzyme with one missed cleavage allowed. The fixed modifications of carbamidomethylation were set as cysteine (Cys), and variable modifications of oxidation as methionine (Met). The peptide tolerance was set as 0.05 Da, and MS/MS tolerance was set as 0.1 Da. The ratio of each protein is given by the geometric mean of the protein ratios measured from all replicates. A Student’s *t*-test was used to analyze the significance of protein geometric ratios, and the Benjamini–Hochberg multiple hypothesis test correction was employed to correct the *p*-values. Differentially expressed proteins (DEPs) were screened out using 1.2 normalized protein geometric ratios at a *p* < 0.05 level with 5% FDR correction.

### 2.5. mRNA and Protein Correlation Analysis

To compare the concordance between transcriptome changes and proteome changes after nano-CaCO_3_ treatment at S4, a correlation analysis was performed based on quantitative and differentially expressed proteins and genes from the aspects of expression and functional enrichment. Additionally, Spearman’s correlation tests were conducted on transcriptome and proteome.

### 2.6. Gene Expression Analysis with Quantitative Real-Time PCR (qRT-PCR)

Gene transcript levels were analyzed using qRT-PCR with a BIO-RAD CFX Connect™ Optics Module (Bio-Rad, Hercules, CA, USA). The cDNA was synthesized from RNA using PrimeScript^®^ RT reagent Kit With gDNA Eraser (TaKaRa, Kusatsu, Japan). *Actin* (JN105299) was used as an internal control in *P. lactiflora* [20]. All gene-specific primers in this study were shown in Table S1, and synthesized by Shanghai Sangon Biological Engineering Technology and Services Co., Ltd. (Shanghai, China). qRT-PCR was performed using the SYBR^®^ Premix Ex Taq™ (Perfect Real Time) (TaKaRa, Japan) and contained 12.5 µL 2 × SYBR Premix Ex Taq™, 2 µL cDNA solution, 2 µL mix solution of target gene primers and 8.5 µL ddH_2_O in a final volume of 25 µL. The amplification was carried out under the following conditions; 50 °C for 2 min followed by an initial denaturation step at 95 °C for 30 s, 40 cycles at 95 °C for 5 s, 51 °C for 15 s, and 72 °C for 30 s. Gene relative expression levels of target genes were calculated by the 2^−^^ΔΔCt^ comparative threshold cycle (Ct) method [21]. The Ct values of the triplicate reactions were gathered using the Bio-Rad CFX Manager V1.6.541.1028 software (Bio-Rad, Hercules, CA, USA).

### 2.7. Metabolome with LC-MS/MS and Data Analysis

The samples used in RNA-seq and iTRAQ were used to perform metabolome analysis. Twenty-five microgram samples were treated with 800 µL of methanol: H_2_O mixture (1:1, v:v) (precooled at −20 °C). The tubes were placed into an ultrasound machine at 60 HZ for 5 min, and then centrifuged for 20 min at 25,000 g and the supernatant was transferred into 2 mL tube. After the above reactions, metabolite analysis was performed by the LC-MS system following the machine orders. Firstly, all chromatographic separations were performed using an ultraperformance liquid chromatography (UPLC) system (Waters, Milford, MA, USA). An ACQUITY UPLC BEH C18 column (100 mm × 2.1 mm, 1.7 µm, Waters) was used for the reversed phase separation. A high-resolution tandem mass spectrometer SYNAPT G2 XS QTOF (Waters) was used to detect metabolites eluted form the column. The Q-TOF was operated in both positive and negative ion modes. For positive ion mode, the capillary and sampling cone voltages were set at 2 kV and 40 V, respectively. For negative ion mode, the capillary and sampling cone voltages were set at 1 kV and 40 V, respectively. The mass spectrometry data were acquired in Centroid MSE mode. The TOF mass range was from 50 to 1200 Da and the scan time was 0.2 s. For the MS/MS detection, all precursors were fragmented using 20 to 40 eV, and the scan time was 0.2 s. During the acquisition, the LE signal was acquired every 3 s to calibrate the mass accuracy. Furthermore, in order to evaluate the stability of the LC-MS during the whole acquisition, a quality CG sample (pool of all samples) was acquired after every 10 samples.

Metabolite identification was accomplished using Progenesis QI 2.2, including peak alignment, peak extraction, homogenization, deconvolution, and compound identification. The data were corrected using the quality CG-based robust LOESS signal correction (QC-RLSC) which was the more effective data correction method in the metabolomic group data analysis. Two sample *t*-test statistics were used for the comparison of metabolite levels to determine the differences between the Control and nano-CaCO_3_ treatment group. The response ratio (a fold change test) was computed for each metabolite to evaluate the extent of differences in pair wise comparison. Principal component analysis (PCA) was based on the correlation matrix (cases = each sample; variables = metabolites). Partial least squares discriminant analysis (PLS-DA) reflected the furthest differences between the groups. Through calculating the variable important for the projection (VIP), it supported the screening of different metabolic by VIP ≥ 1.0. The threshold for significantly differentially expressed metabolites (DEMs) was set at a VIP ≥ 1.0 combined with a fold change ≥ 1.2 or ≤ 0.833 and *q* value < 0.05. The metabolic pathway information was retrieved from the KEGG.

### 2.8. Microstructures Observation and Histological Staining

The microstructures of inflorescence stems were observed by the environmental scanning electron microscopy (SEM) (XL-30 ESEM, Philips, Amsterdam, Holland) and transmission electron microscopy (TEM) (CM100, Philips, Amsterdam, Holland), which were performed as described previously [22]. Histological staining was used to observe cell wall compositions. The fixed inflorescence stems were firstly dehydrated gradually in 50, 70, 90, and 100% ethanol solutions and dehydrated with a xylene and ethanol mixture (1/3, 1/1, 3/1; *v*/*v*) for 30 min each. Moreover, the samples were dehydrated twice with a xylene and chloroform mixture (9/1, *v*/*v*) for 30 min. After infiltration and embedding with paraffin, the sections (8 µm) were obtained using a rotary microtome (RM2245, Leica, New York, NY, USA). Additionally, after paraffin was removed using xylene, the sections were subsequently rehydrated in 100, 95, 80, 50, and 30% ethanol followed by distilled water, and they were finally air dried. For lignin staining, the sections were stained with 2% (*w*/*v*) phloroglucinol for 2 min, and then treated in 18% HCl. Subsequently, the stained sections were observed immediately under a light microscope (CX31RTSF, Olympus, Tokyo, Japan).

### 2.9. Cell Wall Compositions Analysis

Cell wall materials were extracted from inflorescence stems as described previously [23]. X-ray photoelectron spectroscopy (XPS) determination of cell wall materials was performed on a spectrometer (ESCALAB250Xi, Thermofisher Scientific, Waltham, MA, USA) using a set monochromatic radiation A1Kα source with a background pressure of 5 × 10^−10^ mbar. Each analysis consisted of a wide survey scan (pass energy 100 eV, 1.0 eV step size) and a high-resolution scan (pass energy 30 eV, 0.1 eV step size) for component speciation. The takeoff angle was 90° in all measurements. The binding energies of the peaks were determined using the C1s speak at 284.8 eV. The C1s speaks were resolved into five peaks using a peak-fit program. These five peaks were the saturated carbon C1 (C–C), the hydroxyl carbon C2 (C–O), the carbonyl carbon C3 (O–C–O or C=O), the carboxyl carbon C4 (O–C=O), and the carbonate C5. The fitting was performed with 90% Gaussian and 10% Lorentzian curve shapes (28). Neither the relative peak position nor the relative peak width was fixed in the curve-fitting process. Fourier-transform infrared spectroscopy (FTIR) determination of cell wall materials was analyzed by an IR spectrometer (670-IR + 610-IR, Varian, Baltimore, MD, USA) using the KBr pellet technique. The spectrum was taken in the wave number range from 4000 to 400 cm^−1^ with a resolution of 4 cm^−1^ and 32 scans per sample. All spectra were normalized and baseline-corrected with OPUS management software.

### 2.10. Statistical Analysis

All experiments described here were repeated three times arranged in a completely randomized design. The results were analyzed for variance using the SAS/STAT statistical analysis package (version 6.12, SAS Institute, Cary, NC, USA). The data shown in the figures were means ± SDs and the different letters indicated significant differences (*p* < 0.05).

## 3. Results

### 3.1. Morphological Indices and Photosynthetic Characteristics

**Figure 1:**
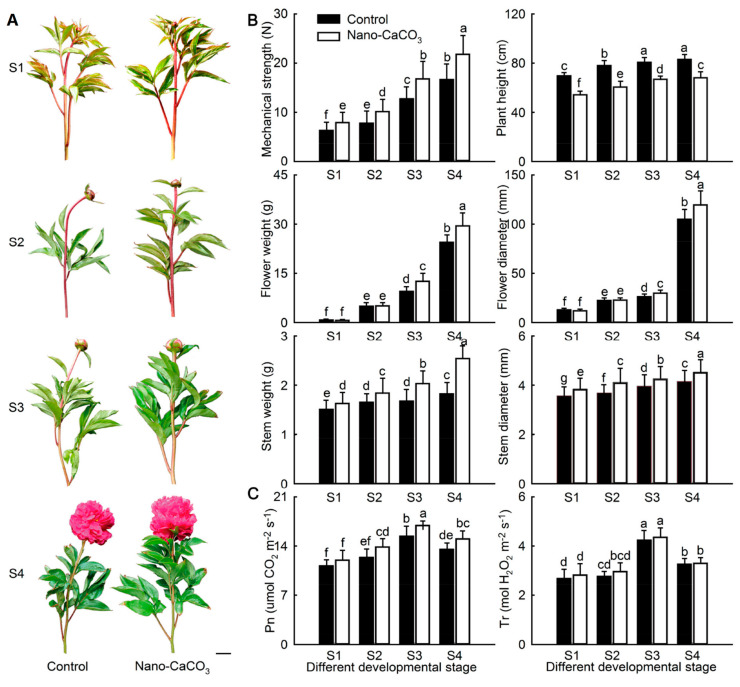
Effects of nano-CaCO_3_ treatment on morphological indices and photosynthetic characteristics of *P. lactiflora* at four developmental stages. (**A**) Photographs of inflorescence stems; bar = 5 cm. (**B**) Morphological indices. (**C**) Photosynthetic characteristics. *Pn*, photosynthesis rate; *Tr*, Transpiration rate. The values represent the means ± SDs, and different letters indicate significant differences (*p* < 0.05). S1, flower-bud stage; S2, pigmented stage; S3, unfold-petal stage; S4, full-flowering stage.

Nano-CaCO_3_ treatment significantly affected the growth and development of *P. lactiflora*. In terms of morphology, the top inflorescence stems of *P. lactiflora* bent during flower development, whereas it became straight and upright after treatment with nano-CaCO_3_ (Figure 1A). Moreover, the mechanical strength of the inflorescence stems showed a gradual increasing tendency. The value of mechanical strength in the nano-CaCO_3_ treatment was significantly higher than that in the Control, especially at S3 and S4, where the strength increased by more than 30%. The same increasing trend was also detected in other morphological indices, including plant height, flower weight and diameter, and inflorescence stem weight and diameter, in both the Control and nano-CaCO_3_ treatment. Compared with the Control, all indices in Nano-CaCO_3_ treatment were increased, except for plant height (Figure 1B). Additionally, both *Pn* and *Tr* increased gradually until S3 and then declined in both Control and nano-CaCO_3_ treatment. *Pn* was higher after nano-CaCO_3_ treatment than in the Control, and a significant increase of greater than 9.88% was attained from S3 to S4. In contrast, although the value of *Tr* after nano-CaCO_3_ treatment was slightly higher than that of the Control, the difference was not significant (Figure 1C). As a result, nano-CaCO_3_ treatment significantly enhanced the mechanical strength of inflorescence stem and increased the photosynthesis of *P. lactiflora*.

### 3.2. Transcriptome Analysis

To elucidate the mechanism underlying the Ca-mediated enhancement of *P. lactiflora* inflorescence stem mechanical strength, we initially performed RNA-seq. The top 12 cm of inflorescence stems were collected at S4 of the Control and nano-CaCO_3_ treatment for RNA isolation. Shotgun libraries were constructed and sequenced. In total, a total of 40.31 Gb sequence data were obtained (NCBI accessions: SRP127132). After quality checks, adapter trimming, and size selection, the clean reads ratio of the Control and nano-CaCO_3_ treatment were 83.48%, 89.89%, and 84.40% and 90.72%, 87.85%, and 86.37%, respectively (Table S2). Then de novo assembly was performed using Trinity and a final high-quality dataset of 107,929 unigenes with an average length of 781 bp and an N50 of 1488 bp was obtained (Table S3). Subsequently, these unigenes were annotated using seven public databases. In total, 61,483 unigenes were annotated, accounting for 56.97% of all unigenes. Among them, 55,138, 46,445, 37,335, 41,032, 22,478, 43,952, and 26,895 unigenes could be annotated to the NR, NT, Swiss-Prot, KEGG, COG, GO databases, respectively, accounting for 51.09, 43.03, 34.83, 38.02, 20.83, 40.72, and 24.92% of the unigenes, respectively.

**Figure 2:**
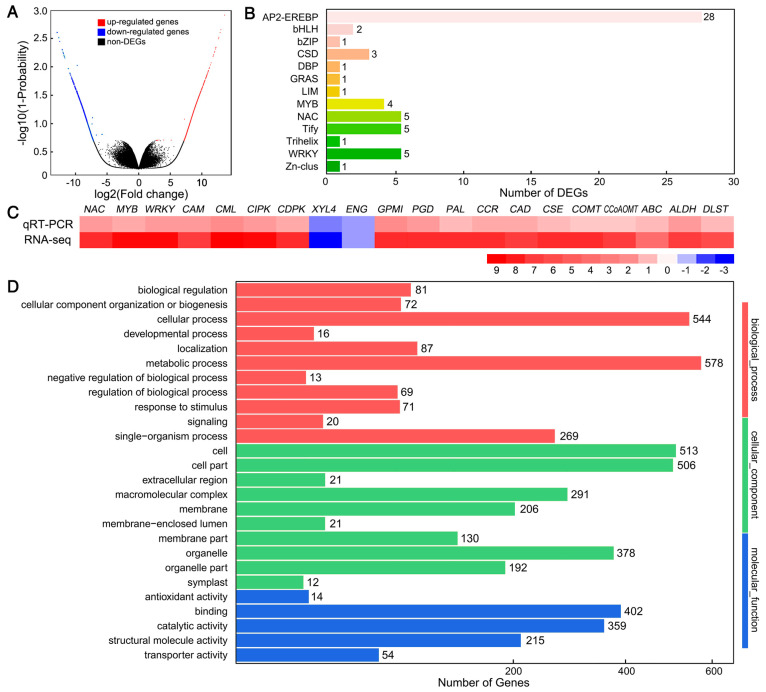
Effects of nano-CaCO_3_ treatment on the transcriptome of *P. lactiflora* inflorescence stems. (**A**) Volcano plot of differentially expressed genes (DEGs). The *X* axis represents log2-transformed fold change, the *Y* axis represents -log10-transformed adjusted *p*-values, the red points represent upregulated DEGs, the blue points represent downregulated DEGs, and the black points represent non-DEGs. (**B**) Transcription factor family classification of DEGs. The *X* axis represents the number of DEGs and the *Y* axis represents the family of transcription factors. (**C**) Heat map of the log2 relative expression levels of the expression results for 20 DEGs in nano-CaCO_3_/Control obtained from RNA-seq and qRT-PCR analysis; *CAM*, Calmodulin gene; *CML*, Calmodulin-like protein gene; *CIPK*, calcineurin B-like protein-interacting protein kinase gene; *CDPK*, calcium-dependent protein kinase gene; XYL4, xylan 1,4-beta-xylosidase gene; *ENG*, endoglucanase gene; *GPMI*, 2,3-bisphosphoglycerate-independent phosphoglycerate mutase gene; *PGD*, 6-phosphogluconate dehydrogenase gene; *PAL*, phenylalanine ammonia-lyase gene; *CCR*, cinnamoyl-CoA reductase gene; *CAD*, cinnamyl-alcohol dehydrogenase gene; *CSE*, caffeoyl shikimate esterase gene; *COMT*, caffeic acid 3-*O*-methyltransferase gene; *CCoAOMT*, caffeoyl-CoA 3-*O*-methyltransferase gene; *ABC*, ABC transporters; *ALDH*, Alcohol dehydrogenase; *DLST*, Dihydrolipoamide succinyltransferase. (**D**) GO classification of DEGs. The *X* axis represents the number of DEGs, the *Y* axis represents the GO terms.

After comparative analysis, a total of 2643 DEGs were obtained, including 1466 upregulated and 1177 downregulated DEGs (Figure 2A). Among these DEGs, there were 11 transcription factor families, and transcription factor enrichment analysis showed a predominance of *AP2-EREBP*, *NAC*, *Tify*, *WRKY*, and *MYB* (Figure 2B). To validate these DEGs, the expression levels of 20 randomly selected DEGs were analyzed by qRT-PCR. We found that the log2 relative expression levels of the results obtained for nano-CaCO_3_/Control from RNA-Seq and qRT-PCR analyses were consistent, and that there was a significant positive correlation (R^2^ = 0.9182) between them, thereby indicating that the RNA-seq results were credible (Figure 2C). To elucidate the functions of these DEGs, they were assigned to GO categories and grouped into the following three main categories: biological process, cellular component, and molecular function (Figure 2D).

Totally, 2643 DEGs were obtained after nano-CaCO_3_ treatment through RNA-seq and the results were credible through qRT-PCR analyses.

### 3.3. Integrative Analysis of Proteome and Transcriptome

Proteome analysis of the same samples assayed by RNA-seq was performed using the iTRAQ method. As a result, 256,853 spectra with 19,326 matching known peptides were obtained, and 5045 proteins were identified. Under nano-CaCO_3_ treatment, 892 DEPs compared to the control were found. Of these, 492 were upregulated and 400 were downregulated (Figure S1A). To further explore the functions of these DEPs, KEGG enrichment analyses were implemented. The 892 DEPs were annotated into 102 KEGG pathways, and were significantly enriched in eight pathways including ribosome, phenylpropanoid biosynthesis, cyanoamino acid metabolism, and so on (Figure S1B).

**Figure 3:**
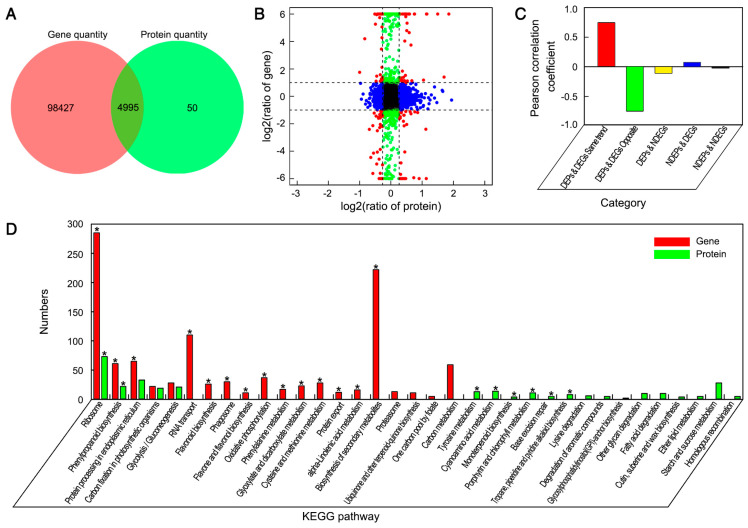
Effects of nano-CaCO_3_ treatment on the proteome of *P. lactiflora* inflorescence stems. (**A**) Venn diagram of the correlation numbers between transcriptome and proteome. The red color represents gene quantity and the green color represents protein quantity. (**B**) Expression ratios from transcriptomic and proteomic profiles based on quantitative proteins and correlated genes. The *X* axis represents log2-transformed fold change of proteins and the Y axis represents log2-transformed fold change of correlated genes; significant expression changes are indicated by different colors: blue points, proteins only; green points, transcripts only; and red points, both. (**C**) Pearson coefficient distribution of the correlation numbers between transcriptome and proteome in five categories. The *X* axis represents category, the *Y* axis represents the Pearson correlation coefficient. (**D**) Pathway enrichment analysis of DEGs and differentially expressed proteins (DEPs). The X axis represents the number of DEGs and DEPs, the *Y* axis represents the pathway name, the red color represents gene and the green represents protein, and * represents DEG and DEP significantly enriched pathways.

**Figure 4:**
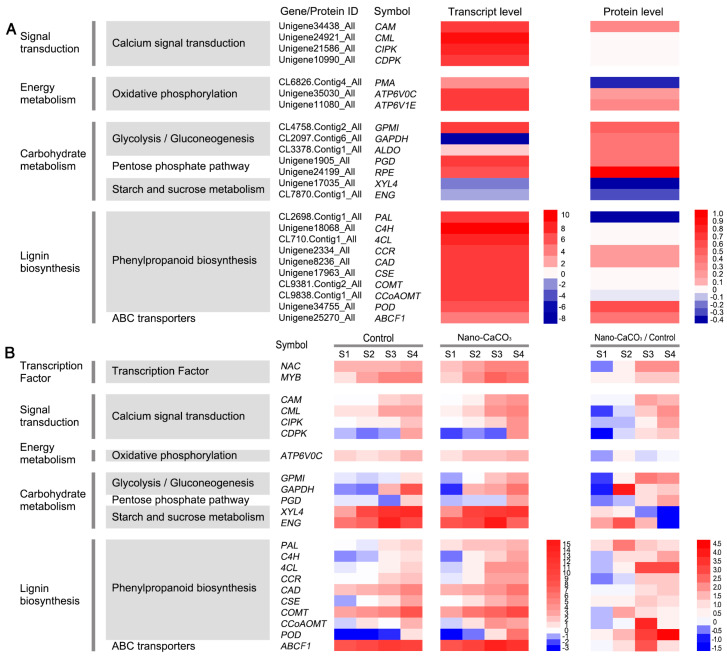
Heat maps of the expression of the genes. (**A**) Heat maps of correlated DEG and DEP expression involved in signal transduction, energy metabolism, carbohydrate metabolism, and lignin biosynthesis, with green indicating a decrease and red indicating an increase. (**B**) Heat maps of the expression of the 22 protein-encoding genes during four developmental stages. The log2 relative expression levels of 22 protein-encoding genes in the Control, nano-CaCO_3_ treatment, with red color indicating a higher expression level and blue color indicating a lower expression level. The ratio of relative expression levels of 22 protein-encoding genes in nano-CaCO_3_/Control, with red color indicating an increase and blue indicating a decrease. *CAM*, Calmodulin gene; *CML*, Calmodulin-like protein gene; *CIPK*, calcineurin B-like protein-interacting protein kinase gene; *CDPK*, calcium-dependent protein kinase gene; *ATP6V0C*, V-type H^+^-transporting ATPase 16kDa proteolipid subunit gene; *GPMI*, 2,3-bisphosphoglycerate-independent phosphoglycerate mutase gene; *GAPDH*, glyceraldehyde 4-phosphate dehydrogenase gene; *PGD*, 6-phosphogluconate dehydrogenase gene; *XYL4*, xylan 1,4-beta-xylosidase gene; *ENG*, endoglucanase gene; *PAL*, phenylalanine ammonia-lyase gene; *C4H*, cinnamate 4-hydroxylase gene; *C4L*, 4-coumarate-CoA ligase gene; *CCR*, cinnamoyl-CoA reductase gene; *CAD*, cinnamyl-alcohol dehydrogenase gene; *CSE*, caffeoyl shikimate esterase gene; *COMT*, caffeic acid 3-*O*-methyltransferase gene; *CCoAOMT*, caffeoyl-CoA 3-*O*-methyltransferase gene; *POD*, peroxidase gene; *ABCF1*, ABC subfamily F member 1. S1, flower-bud stage; S2, pigmented stage; S3, unfold-petal stage; S4, full-flowering stage.

The concordance analysis of expression changes in genes and proteins was conducted. In this study, more than 99% of proteins identified in the proteome analysis were also covered by the transcriptome analysis, whereas less than 1% of proteins were identified only by iTRAQ (Figure 3A). The fold expression levels of proteins were correlated with their respective mRNA values in nano-CaCO_3_/Control. From differentially expressed type, the number of correlations between 2643 DEGs and 892 DEPs was 152 (Figure 3B). When expression changes in the transcript and protein levels were compared, a poor correlation between the levels of quantitative proteins and those of their gene transcripts was observed, whereas there was a relatively high correlation between DEGs and DEPs (Figure 3C). Subsequently, KEGG enrichment analysis of the DEGs and DEPs was performed, and the top 20 KEGG pathways of DEGs and DEPs were shown in Figure 3D and the detailed data were presented in Table S4. As a result, DEGs were accordingly found to be significantly enriched in a total of 14 pathways, whereas DEPs were significantly enriched in eight pathways. By contrast, only phenylpropanoid biosynthesis and ribosome were significantly enriched at both the transcript and protein levels. Phenylpropanoid biosynthesis is an important pathway for lignin biosynthesis. In this pathway, nine genes and the correlated proteins had been detected, namely, phenylalanine ammonia-lyase gene (*PAL*), cinnamate 4-hydroxylase gene (*C4H*), 4-coumarate-CoA ligase gene (*4CL*), cinnamoyl-CoA reductase gene (*CCR*), cinnamyl-alcohol dehydrogenase gene (*CAD*), caffeoyl shikimate esterase gene (*CSE*), caffeic acid 3-*O*-methyltransferase gene (*COMT*), caffeoyl-CoA 3-*O*-methyltransferase gene (*CCoAOMT*), and peroxidase gene (*POD*). The expression patterns of these genes were shown in Figure 4A, among which, *C4H*, *4CL*, *CSE*, *COMT*, and *CCoAOMT* were significantly upregulated at the transcript level, although there was no significant differences were detected at the protein level. *PAL*, *CCR*, *CAD*, and *POD* were significantly differentially expressed at both transcript and protein levels. Among these, *CCR*, *CAD*, and *POD* were all upregulated at the transcript and protein levels, whereas *PAL* showed the opposite expression pattern. Additionally, the Ca^2+^ sensors, including calmodulin gene (*CAM*), CAM-like protein gene (*CML*), calcineurin B-like protein-interacting protein kinase gene (*CIPK*), and calcium-dependent protein kinase gene (*CDPK*), were also differentially expressed at the transcript level. *CAM* was also significantly activated at the protein level in response to nano-CaCO_3_ treatment. In addition to the correlated genes and proteins involved in phenylpropanoid biosynthesis and calcium signal transduction, we also screened 11 correlated DEGs and DEPs from 152 associated DEPs and DEGs referred to previous studies, including H^+^-transporting ATPase gene (*PMA*), V-type H^+^-transporting ATPase 16kDa proteolipid subunit gene (*ATP6V0C*), and V-type H^+^-transporting ATPase subunit E gene (*ATP6V1E*) in oxidative phosphorylation; 2,3-bisphosphoglycerate-independent phosphoglycerate mutase gene (*GPMI*), glyceraldehyde 4-phosphate dehydrogenase gene (*GAPDH*), and fructose-bisphosphate aldolase gene (*ALDO*) in glycolysis; 6-phosphogluconate dehydrogenase gene (*PGD*) and ribulose-phosphate 3-epimerase gene (*RPE*) in pentose phosphate pathway; xylan 1,4-beta-xylosidase gene (*XYL4*) and endoglucanase gene (*ENG*) in starch and sucrose metabolism; and ABC subfamily F member 1 (*ABCF1*) in ABC transporters (Table S5). The expression patterns of these genes could be divided into the following categories. *ATP6V0C*, *ATP6V1E*, *GPMI*, *ALDO*, *PGD*, *RPE*, and *ABCF1* were all increased at both transcript and protein levels; *XYL4* and *ENG* were all decreased at both transcript and protein levels; *PMA* was increased at the transcript level but showed a decreased protein level; whereas *GAPDH* was increased at the protein level but exhibited a decreased transcript level (Figure 4A).

The expression patterns of most selected genes showed a rising trend from S1 to S4 in both Control and nano-CaCO_3_ treatment. The exceptions to this trend were the expressions of *NAC*, *CDPK*, and *PGD* in the Control, which showed an initial decrease followed by an increase; the expression of *CML*, *ATP6V0C*, *PAL*, and *POD* in the Control and *PGD* in nano-CaCO_3_ treatment was initially maintained at a steady rate and then showed an increasing trend; in both Control and nano-CaCO_3_ treatment, the expression of *ENG* and *ABCF1* expression initially increased from S1 to S3 and then decreased at S4 (Figure 4B). Compared with the Control, the expression levels of these genes were higher in nano-CaCO_3_ treatment during the development of inflorescence stems, particularly *GAPDH* at S2; *NAC*, *CAM*, *GPMI*, *4CL*, *CCoAOMT*, *POD*, and *ABCF1* at S3; and *NAC*, *CML*, *4CL*, and *POD* at S4. However, at S1, the expression of a half of these genes was lower than that of the Control. Additionally, *XYL4* showed a lower expression at S3 and S4, and *ENG* showed a lower expression at S4 (Figure 4B).

In general, 892 DEPs were obtained after Nano-CaCO_3_ treatment, and 152 were coregulated at both the proteomic and transcriptomic levels, which were both enriched in phenylpropanoid biosynthesis and ribosome. Twenty-four DEPs related to the secondary cell wall that are involved in signal transduction, energy metabolism, carbohydrate metabolism, and lignin biosynthesis were screened, most of which were upregulated after nano-CaCO_3_ treatment during inflorescence stem development.

### 3.4. Integrative Analysis of Metabolome, Proteome, and Transcriptome

**Figure 5:**
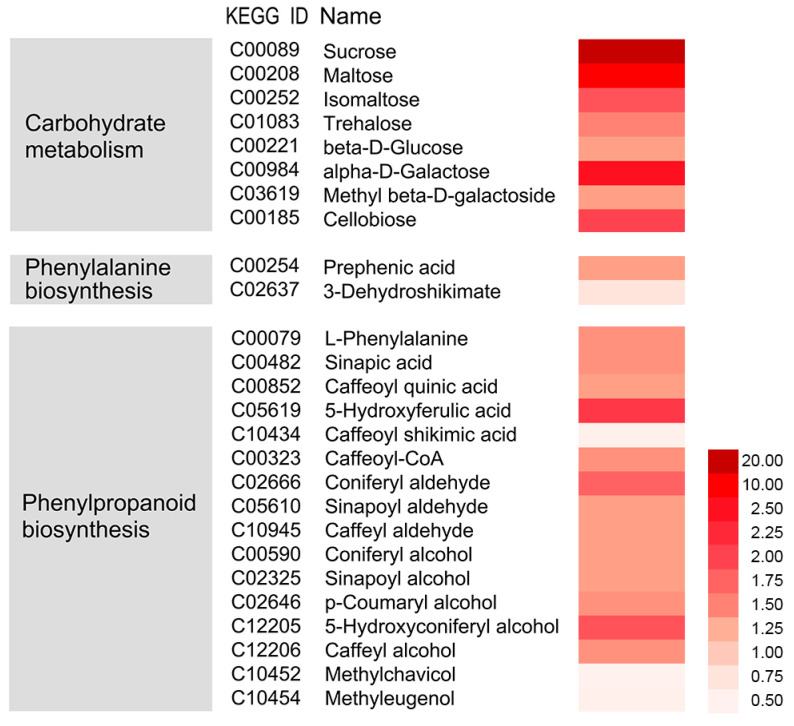
Effects of nano-CaCO_3_ treatment on the metabolome of *P. lactiflora* inflorescence stems. Heat map of log2 relative levels of the main metabolites in nano-CaCO_3_/Control involved in carbohydrate metabolism, phenylalanine biosynthesis, and phenylpropanoid biosynthesis. The dark red and light red colors indicate increased and decreased metabolites, respectively.

**Figure 6:**
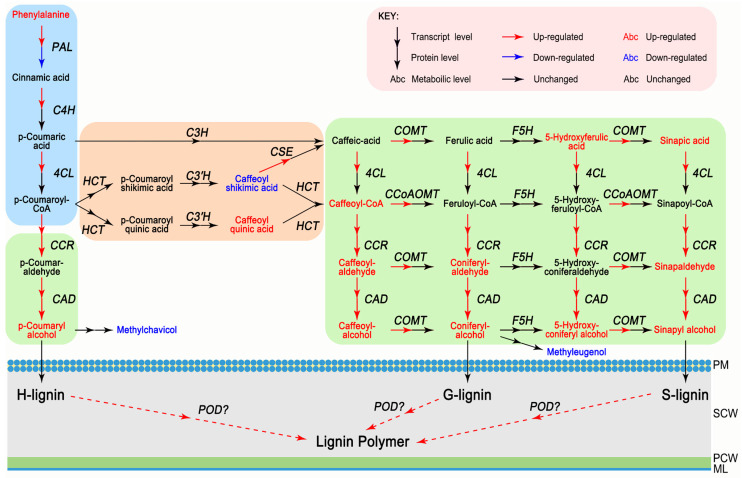
Integration of nano-CaCO_3_ treatment-induced *P. lactiflora* inflorescence stems mRNAs, proteins, and metabolites in the phenylpropanoid biosynthesis pathway. PM, plasma membrane; SCW, secondary cell wall; PCW, primary cell wall; ML, middle lamella. *PAL*, phenylalanine ammonia-lyase gene; *C4H*, cinnamate 4-hydroxylase gene; *C4L*, 4-coumarate-CoA ligase gene; *CCR*, cinnamoyl-CoA reductase gene; *CAD*, cinnamyl-alcohol dehydrogenase gene; *HCT*, *p*-hydroxycinnamoyl-CoA:quinate/shikimate *p*-hydroxycinnamoyltransferase; *C3H*, *p*-coumarate 3-hydroxylase; *C3′H*, *p*-coumarate 3′-hydroxylase; *CSE*, caffeoyl shikimate esterase gene; *COMT*, caffeic acid 3-*O*-methyltransferase gene; *F5H*, ferulate 5-hydroxylase; *CCoAOMT*, caffeoyl-CoA 3-*O*-methyltransferase gene; *POD*, peroxidase gene.

The effects of the nano-CaCO_3_ treatment on transcriptomic and proteomic changes discussed in previous sections were further validated using metabolome analysis with LC-MS/MS. Nano-CaCO_3_ treatment had a considerable effect on cellular metabolites, and 26 differential metabolites were screened with their detailed data listed in Table S6. Sixteen of the differential metabolites were detected in phenylpropanoid biosynthesis pathway, whereas the remainder were distributed in various metabolic pathways, including those of indole-3-acetic acid, gibberellin A1, jasmonic acid, and salicylic acid involved in plant hormone; sucrose, maltose, and isomaltose involved in carbohydrate metabolism; beta-d-glucose and alpha-d-galactose related to hemicellulose synthesis; cellobiose related to cellulose metabolism; and 3-dehydroshikimate and prephenic acid involved in phenylalanine biosynthesis. As shown in Figure 5, with the exception of caffeoyl shikimic acid, methylchavicol, and methyleugenol, there was a significant increase in the accumulation of these metabolites. The integration of nano-CaCO_3_ treatment-induced mRNAs, proteins, and metabolites is shown in Figure 6. The result shows that the expression of *C4H*, *4CL*, *CSE*, *COMT*, and *CCoAOMT* increased, whereas there was no change in the level of protein expression. Additionally, some genes, such as *PAL*, were induced, whereas the corresponding proteins were inhibited. In contrast, in some instances—including *CCR*, *CAD*, and *POD*—both genes and the corresponding proteins were induced. Among these genes, *CCR*, *CAD*, and *POD* catalyze the synthesis of H-lignin, G-lignin, and S-lignin. Furthermore, accumulation of the precursors of H-lignin, G-lignin, and S-lignin, such as *p*-coumaryl alcohol, caffeoyl-alcohol, coniferyl-alcohol, and sinapyl alcohol, all increased. The accumulation of methylchaicol and methyleugenol, although having the same precursors as H-lignin and G-lignin, decreased after nano-CaCO_3_ treatment. In summary, 26 differential metabolites were screened based on the transcriptomic and proteomic analysis, and 16 of them were detected in phenylpropanoid biosynthesis pathway that were mostly upregulated.

### 3.5. Microstructures

**Figure 7:**
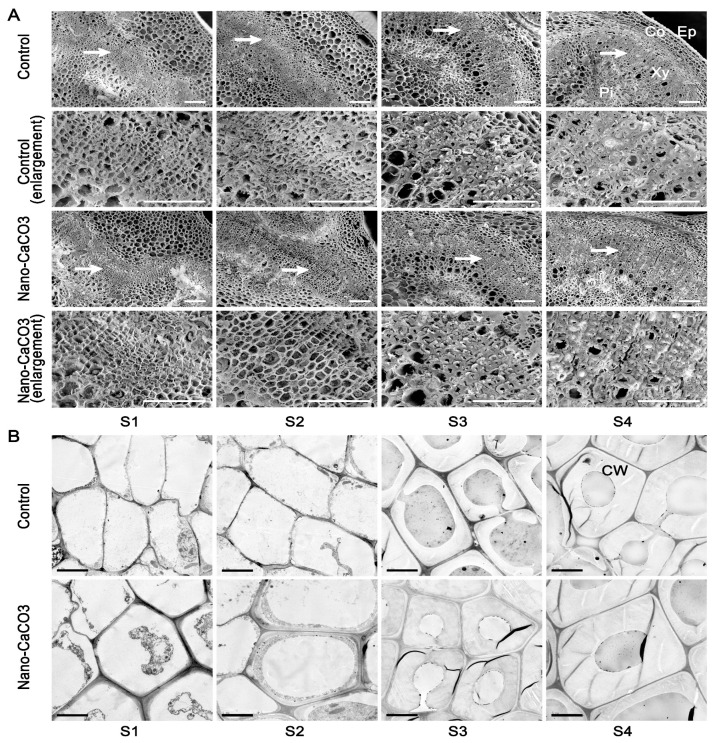
Effects of nano-CaCO_3_ treatment on the microstructures of *P. lactiflora* inflorescence stems at four developmental stages. (**A**) SEM. Sections of inflorescence stem in Control and nano-CaCO_3_ treatment. Micrographs of partial enlargement of regions are marked by the arrow. Ep, epidermis; Co, cortex; Pi, pith; Xy, xylem. Bars, 100 µm. (**B**) TEM. Sections of inflorescence stem in Control and nano-CaCO_3_ treatment. CW, cell wall. Bars, 5 µm. S1, flower-bud stage; S2, pigmented stage; S3, unfold-petal stage; S4, full-flowering stage.

We also observed the microstructures of the inflorescence stems in both the Control and nano-CaCO3-treated groups. Scanning electron micrographs revealed that various parts had formed at S1, i.e., epidermis, cortex, and central cylinder; there were no significant differences between the Control and nano-CaCO_3_ treatment. However, as the inflorescence stems continue to develop, certain changes occurred. For example, cells became more closely arranged, and the sclerenchyma cell walls thickened, and these changes were particularly evident at S4. When compared with the Control, the thickness of the sclerenchyma cell walls and the number of thickened cell layers under nano-CaCO_3_ treatment were significantly enhanced (Figure 7A). In order to better observe the thickness of the sclerenchyma cell walls, transmission electron microscopy was performed, and the observations were consistent with those using scanning electron microscopy, i.e., the sclerenchyma cell walls under Nano-CaCO_3_ treatment were apparently thicker than those in the Control (Figure 7B). Measured using a micrometer, the thickness of the sclerenchyma cell walls under nano-CaCO_3_ treatment increased significantly compared with that in the Control from S2 to S4 (257.87%), with a staged increase of 20.19% and 25.22% from S2 to S3 and S3 to S4, respectively. As a result, nano-CaCO_3_ treatment significantly increased the thickness of the sclerenchyma cell walls and the number of the thickened cell layers.

### 3.6. Cell Wall Compositions

**Figure 8:**
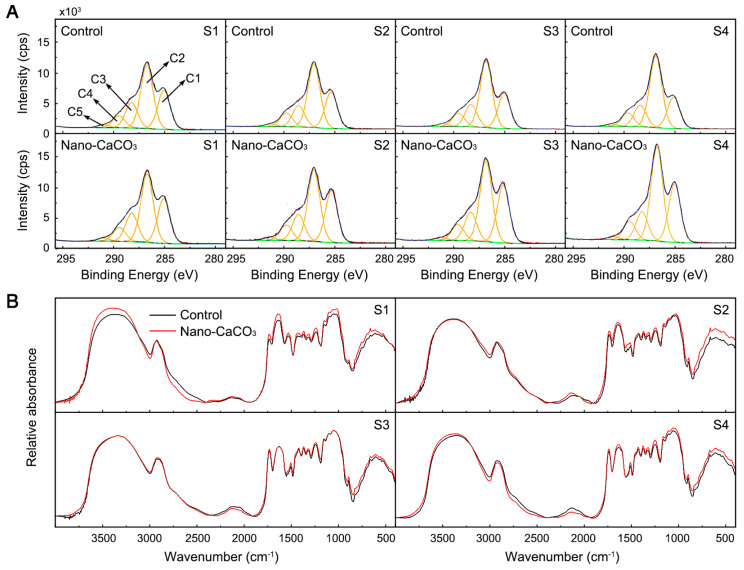
Effects of nano-CaCO_3_ treatment on the cell wall compositions of *P. lactiflora* inflorescence stems. (**A**) Deconvoluted high-resolution XPS C_1s_ spectrum of cell wall. (**B**) Absorption FTIR spectra of cell wall in the 4000–400 cm^−1^ region. S1, flower-bud stage; S2, pigmented stage; S3, unfold-petal stage; and S4, full-flowering stage.

To verify whether lignin in the thickened cell walls plays a decisive role in the mechanical strength of *P. lactiflora* inflorescence stems, cell wall compositions were determined. Firstly, we determined the elemental composition of cell walls using XPS. The elemental composition of the cell wall materials that resulted from the integration of C_1s_, O_1s_, N_1s_, and Ca_2p_ peaks from the wide-scan spectrum can be seen in Table 1. With the exception of S1, nano-CaCO_3_ treatment promoted an increase in N and Ca concentrations in the cell walls of the inflorescence stems, and concentration differences reached a significant level at S3. In contrast, no significant differences were detected between the Control and nano-CaCO_3_ treatment with respect to C and O. The XPS peaks corresponding to C were analyzed at high resolution and deconvoluted to assess the contributions from each component [24]. Figure 8A shows the deconvoluted XPS spectra of the C1s emission line for the cell walls of Control and nano-CaCO3-treated plants. Regardless of treatment, the carbon peak (C_1s_) fitted five components: carbon bound only to carbon or hydrogen, C–(C,H), at ~284.80 eV (C1); carbon singly bound to oxygen or nitrogen, C–(O,N), at ~286.55 eV (C2); carbon doubly bonded to oxygen (C=O) or singly bonded to two oxygen atoms (O–C–O), at ~288.03 eV (C3); carbon attributable to carboxylic functions, COOR, at ~289.26 eV (C4); and carbon attributable to carbonate functions, RCOO, at ~289.88 eV (C5). The contributions of C2 to the C_1s_ emission line on the XPS spectra were the highest, followed by that of C1, C3, C4, and C5 regardless of the Control or nano-CaCO_3_ treatment (Table 2). After Nano-CaCO_3_ treatment, the relative concentrations of C1 and C2 were slightly increased, and the relative concentrations of C3 and C4 were slightly decreased. With the exception of C5 at S4, the nano-CaCO_3_ treatment induced no significant changes in the relative concentrations of these five components of the C_1s_ emission line.

Subsequently, components and possible cross-links of each fraction were identified by FTIR microspectroscopy after chemical extraction of inflorescence stem cell walls (Figure 8B): the vibration of chemical bonds absorbs radiation in the IR region between 4000 and 400 cm^−1^ and each functional group in a molecule has its characteristic absorption frequencies in the IR spectrum. Functional groups were assigned to spectral peaks as follows; 1740 cm^−1^ (carbonyl C = O), 1640 cm^−1^ (amide I C = O), 1510 cm^−1^ (aromatic skeletal vibrations), 1465 cm^−1^, 1425 cm^−1^, 1325 cm^−1^ (lignin), 1375 cm^−1^ (CH band), 1245 cm^−1^ (amide III in protein), and 1160 cm^−1^, 1107 cm^−1^, 1060 cm^−1^, and 899 cm^−1^ (CHO) [25,26]. The spectra obtained for the Control and nano-CaCO_3_ treatment were generally similar, although there were some quantitative differences in the characteristic regions, indicating the differences in the cell wall compositions. For example, the vibrations at 1640, 1510, 1465, 1425, and 1325 cm^−1^, which were characteristics of lignin or lignin-like structures, were higher in the spectra obtained for nano-CaCO_3_ treatment.

As a whole, nano-CaCO_3_ treatment promoted an increase in Ca concentrations and in contents of chemical groups which respond to lignin or lignin-like structures in the cell walls of the inflorescence stems.

### 3.7. Histochemical Staining

**Figure 9:**
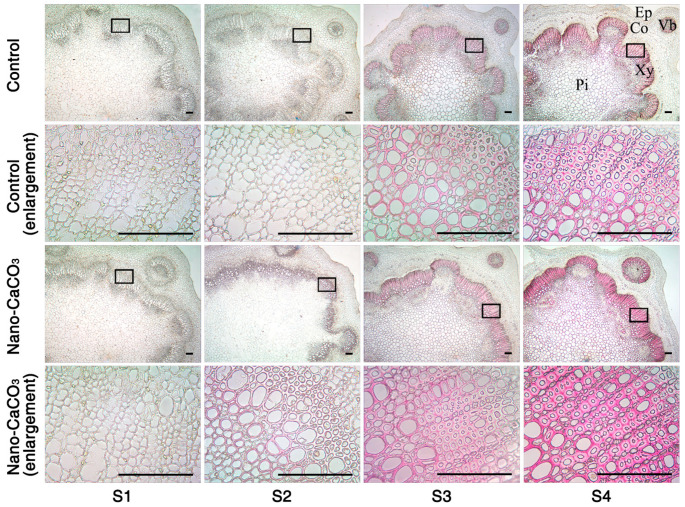
Histochemical staining of lignin in *Paeonia lactiflora* inflorescence stems subjected to Control and Nano-CaCO_3_ treatment at four developmental stages. Transverse sections of 8-µm thickness were stained with phloroglucinol-HCl, which selectively stains lignified cell walls. Sections of inflorescence stem in Control and Nano-CaCO_3_ treatment. Micrographs of partial enlargement of regions are marked by the black box. Ep, epidermis; Co, cortex; Vb, vascular bundle; Pi, pith; Xy, xylem. Bars, 150 µm. S1, flower-bud stage; S2, pigmented stage; S3, unfold-petal stage; and S4, full-flowering stage.

Histochemical staining was used to further examine the alterations in lignin within the thickened cell walls. Wiesner staining using phloroglucinol-HCl is known to react with cinnamaldehyde residues in lignin, and the color intensity is consistent with the total lignin content (3). Between the Control and the nano-CaCO3-treated groups, there were obvious color differences during the development of inflorescence stems, particularly in the sclerenchyma cells and vascular bundle regions (Figure 9). At S1, the transverse sections were virtually unstained, whereas red staining gradually appeared from S2 to S4. The sclerenchyma cells and vascular bundle regions of the nano-CaCO3-treated stems were stained dark red, whereas they were light red in the Control, notably at S4. In short, nano-CaCO_3_ treatment significantly increased the lignin deposition in the sclerenchyma cells and vascular bundle regions of inflorescence stem.

## 4. Discussion

As one of the essential nutrients in plants, Ca^2+^ has long been recognized as a second messenger in signal transduction [27]. It plays an important physiological role in regulating plant cell functions (such as cell elongation and division), cell membrane integrity, nitrogen, carbohydrate, and active oxygen metabolism [28] that can affect plant photosynthesis [29], fruit quality [30,31], resistance to stresses [32,33], and stem mechanical strength [9]. With respect to mechanical strength, treatment with exogenous Ca had shown to delay stem bending in *G. jamesonii* [9], and preharvest spraying with 4% calcium acetate or calcium chloride had been observed to promote a marked increase in the mechanical strength of *P. lactiflora* inflorescence stems [23,34]. In the present study, treatment with 2% nano-CaCO_3_ was found to have a similar effect on the mechanical strength of *P. lactiflora* inflorescence stems, with an observed increase of 30.73%. In plants, stem mechanical strength is related to certain morphological indices, including plant height, stem diameter and stem weight, etc. [35,36]. In *P. lactiflora*, the diameter of the inflorescence stem was a direct indicator that could be used in estimating mechanical strength [16], and a significant increase in inflorescence stem diameter had been also found with preharvest spraying of 4% calcium acetate or calcium chloride [23,34]. In the present study, treatment with 2% nano-CaCO_3_ has resulted in a 9.26% increase in *P. lactiflora* inflorescence stem diameter, whereas *P. lactiflora* plant height was significantly reduced by 17.72%, which was consistent with the result obtained for *P. lactiflora* cv. ‘Yinxianxiuhongpao’ treated with 4% calcium acetate [34]. Moreover, inflorescence stem weight, flower weight, and flower diameter were also increased by 38.77, 20.60, and 13.75%, respectively, after nano-CaCO_3_ treatment. These results indicated that nano-CaCO_3_ treatment could not only improve the mechanical strength of inflorescence stems, but also enhance flower quality in *P. lactiflora*. Additionally, nano-CaCO_3_ treatment also enhanced photosynthesis in *P. lactiflora*, which was consistent with the findings of a previous study [23]. And the improvement of flower quality might be due to the enhancement of photosynthesis leading to the increase of photosynthetic products, which promoted the reproductive growth of plants. Therefore, nano-CaCO_3_ could be widely used in *P. lactiflora* cut-flowers to improve the mechanical strength of inflorescence stems, enhance cut flower quality, and photosynthesis.

The mechanical strength of plant stems is mainly determined by the strength of its secondary cell wall [37]. It had been previously reported that through the course of *P. lactiflora* inflorescence stems, the inner vascular bundles began to emerge, the cells became more closely arranged, and sclerenchyma cell walls thickened [16]; these were also observed in the present study. Following nano-CaCO_3_ treatment, we observed a significant increase in the number of thickened cell layers, particularly at S3 and S4, where the numbers were up to 1.75 and 1.56 times higher than the Control group. Moreover, the thickness of sclerenchyma cell walls was also enhanced by 20 to 25% after nano-CaCO_3_ treatment. These results aligned well with the findings of Rojas et al. [38] showing that Ca treatment increased the cell wall thickness and cell area. Furthermore, it had previously been observed that in contrast to *flexible culm 1* (*FC1*) [2], *brittle culm 5* (*BC5*) [39], and *BC12* [40] *O. sativa* mutants, there was an increase in the thickness of sclerenchyma cell walls in wild type plants. Thus, the mechanical strength of *P. lactiflora* inflorescence stems was mostly related to changes in the sclerenchyma cell walls, and that the enhancement of the inflorescence stem diameter and mechanical strength following nano-CaCO_3_ treatment might be attributed to an increase in the thickness of the sclerenchyma cell walls and the number of thickened cell layers.

Secondary cell walls are mainly composed of lignin, polysaccharides cellulose, and hemicelluloses, and the assembly of the complex secondary cell wall structure is dependent of the coordinated interplay of several different biosynthetic and transport pathways [41]. Lignin is the major component of the secondary cell walls and provides compression strength to the walls. Chemically, lignin is a heterogeneous and complex polymer largely derived from three types of hydroxycinnamoyl alcohol monomers, namely the monolignols p-coumaryl alcohol, coniferyl alcohol, and sinapyl alcohol, which are commonly referred to as H-lignin, G-lignin, and S-lignin, respectively [42]. These monolignols are produced in the cytoplasm through phenylpropanoid biosynthesis pathway and are conveyed to the secondary cell walls by specific transporters such as *ABCF1* [43] and polymerized to lignin by class III *POD* [44]. Numerous studies of stem characteristics in *O. sativa FC1* mutants [2], *C. morifolium* [45], sudangrass (*Sorghum sudanense* Stapf.) [46], and kodo millet (*Paspalum scrobiculatum* L.) [36] have demonstrated that a decrease in lignin content led to a reduction in mechanical strength. In the present study, we identified a lignin biosynthesis pathway, which was enriched for 10 DEGs (*PAL*, *C4H*, *4CL*, *CCR*, *CAD*, *CSE*, *COMT*, *CCoAOMT*, *POD*, and *ABCF1*), and five DEPs encoded by *PAL*, *CCR*, *CAD*, *POD*, and *ABCF1*. These all played an important role in lignin biosynthesis and plant development [47]. We also found that the expression levels of these 10 DEGs were all significantly upregulated after the nano-CaCO_3_ treatment, particularly at S4. At the protein level, *CCR*, *CAD*, *POD*, and *ABCF1* were significantly upregulated by nano-CaCO_3_ treatment; whereas *PAL* was downregulated at S4, which induced lignin accumulation in *P. lactiflora* inflorescence stems. These results were further verified by metabolome analysis, which showed that the accumulation of metabolites involved in monolignols biosynthesis increased in response to nano-CaCO_3_ treatment, including the starting product phenylalanine, the intermediate product caffeoyl-CoA, and the precursors coniferyl-alcohol and sinapyl-alcohol. Moreover, the spectra obtained from the XPS and FTIR analyses also revealed an increase in the relative amount of lignin in the cell walls of the inflorescence stems treated with nano-CaCO_3_. Consistently, histochemical staining with phloroglucinol-HCl also revealed that the lignin content was significantly altered in sclerenchyma cell walls after nano-CaCO_3_ treatment. Collectively, these results suggest that lignin played an important role in the mechanical strength of *P. lactiflora* inflorescence stems, which was in line with the finding of previous study [16], and that nano-CaCO_3_-mediated enhancement of inflorescence stem mechanical strength could mainly be attributed to an increase in lignin accumulation.

In plants, countless Ca-binding proteins serve as Ca^2+^ sensors to decode complex signal-specific Ca signals, and these proteins can be classified into following three categories: *CaM*/*CML*, *CDPK*, and *CBL-CIPK* [48]. The Ca^2+^ sensors induce a physiological response to a signal through regulating downstream events after sensing changes in the concentration changes of cellular Ca^2+^ [49]. In the present study, a higher concentration of Ca was detected in *P. lactiflora* inflorescence stems following nano-CaCO_3_ treatment, and four DEGs (*CAM*, *CML*, *CIPK*, and *CDPK*) involved in calcium signal transduction were identified by RNA-seq, all of which were upregulated at S4 in response to nano-CaCO_3_ treatment. Besides, *CAM* was also upregulated at the protein level at S4. These results were consistent with the findings of a previous study in which it was demonstrated Ca increased the expression levels of Ca^2+^ sensors [50], thereby indicating that nano-CaCO_3_ treatment indeed induced calcium signal transduction. In terms of energy metabolism, *PMA*, *ATP6V0C*, and *ATP6V1E* were all differentially expressed and upregulated at both transcriptional and protein levels, with the exception of *PMA* at the protein level. In *G. jamesonii*, the CAM content and H^+^-ATPase and Ca^2+^-ATPase activities were shown to be increased in the readily bending stems treated with calcium chloride, and the mechanism of Ca^2+^-CAM messenger system, which is involved in the control of stem bending, was enhanced [11]. On the basis of these observations, we speculated that nano-CaCO_3_-mediated enhancement of the mechanical strength of *P. lactiflora* inflorescence stems might be attributable to a mechanism associated with the Ca^2+^ messenger system, and particularly the Ca^2+^-CAM messenger system.

Moreover, a total of 58 DEGs classified in 11 transcription factor families were identified in this study, which were enriched in the *AP2-EREBP*, *NAC*, *WRKY*, *Tify*, and *MYB* families. Among these, *NAC* [51,52] and *MYB* families [52,53] family members comprise the vast majority of factors implicated in regulating secondary cell wall biosynthesis. For example, transcription factors *MYB20*, *MYB69*, *MYB79*, *MYB85*, *MYB58*, and *MYB63* function in the control of lignin biosynthetic genes [54,55,56]. Furthermore, members of these two families played roles as CAM-binding transcription factors [49]. In the present study, both *NAC* and *MYB* transcription factors were differentially expressed in response to nano-CaCO_3_ treatment, and their expression levels were higher under Nano-CaCO_3_ treatment, which indicated that calcium-binding proteins might upregulate *NAC* and *MYB* transcription factors, thereby inducing secondary cell wall biosynthesis.

## 5. Conclusions

**Figure 10:**
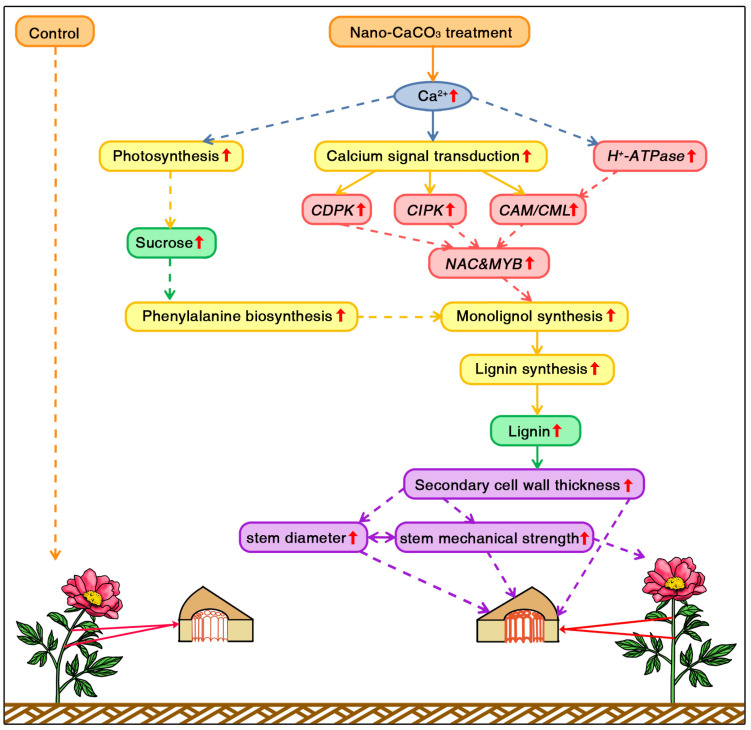
Proposed pathway for calcium-mediated enhancement of the mechanical strength of *P. lactiflora* inflorescence stems. *CDPK*, calcium-dependent protein kinase gene; *CIPK*, calcineurin B-like protein-interacting protein kinase gene; *CAM*, calmodulin gene; *CML*, CAM-like protein gene.

On the basis of the aforementioned observation, we propose a pathway whereby Ca enhanced the mechanical strength of *P. lactiflora* inflorescence stems (Figure 10). Nano-CaCO_3_ treatment increased Ca^2+^ concentrations, and an increase in Ca^2+^ in leaves enhanced plant photosynthesis leading to sucrose accumulation, which laid the material foundation for cell wall thickening.

An increase in the Ca^2+^ concentration in inflorescence stem cell walls induced calcium signal transduction (*CAM*/*CML*, *CIPK*, and *CDPK*), followed in turn by the activation of downstream calcium-binding transcription factors belonging to *NAC* and *MYB* families. As *NAC* and *MYB* members comprised the vast majority factors involved in regulating secondary cell wall biosynthesis, activation of these factors would presumably promote thickening of secondary cell walls by enhancing the biosynthesis of monolignols. For monolignol biosynthesis, the expressions of monolignol biosynthetic genes (*PAL*, *C4H*, *4CL*, *CCR*, *CAD*, *CSE*, *COMT*, and *CCoAOMT*) were significantly upregulated and accumulation of monolignols precursor and lignin increased in the secondary cell wall components. Furthermore, sucrose induced the accumulation of the monolignol substrate phenylalanine, which also enhanced monolignol biosynthesis. Collectively, these processes resulted in an increase in lignin accumulation, and lignin accumulation was associated with an increase in secondary cell wall thickness, which led to an enhancement of inflorescence stem diameter and mechanical strength. These results would contribute to gaining a more comprehensive understanding of the effect of Ca application on *P. lactiflora* cut-flowers.

## Figures and Tables

**Table 1:** Elemental composition of cell walls in *P. lactiflora* inflorescence stems using X-ray photoelectron spectroscopy (XPS).

Stage	Treatment	Element (%)			
		C	O	N	Ca
S1	Control	60.73 ± 0.38 ^a^	33.01 ± 0.21 ^b^	5.90 ± 0.11 ^a^	0.36 ± 0.05 ^e^
	Nano-CaCO_3_	59.72 ± 0.76 ^a^	34.22 ± 0.67 ^ab^	5.69 ± 0.11 ^a^	0.38 ± 0.01 ^e^
S2	Control	61.09 ± 1.13 ^a^	34.31 ± 1.07 ^ab^	4.18 ± 0.35 ^b^	0.42 ± 0.01 ^d^
	Nano-CaCO_3_	61.03 ± 0.76 ^a^	34.25 ± 0.84 ^ab^	4.29 ± 0.11 ^b^	0.43 ± 0.02 ^d^
S3	Control	61.67 ± 1.54 ^a^	34.46 ± 1.26 ^ab^	3.36 ± 0.30 ^d^	0.52 ± 0.03 ^c^
	Nano-CaCO_3_	61.78 ± 0.54 ^a^	33.97 ± 0.55 ^ab^	3.65 ± 0.09 ^c^	0.60 ± 0.04 ^b^
S4	Control	61.83 ± 2.23 ^a^	35.00 ± 1.94 ^a^	2.62 ± 0.26 ^e^	0.56 ± 0.06 ^a^
	Nano-CaCO_3_	61.89 ± 0.54 ^a^	34.37 ± 0.55 ^ab^	3.11 ± 0.04 ^d^	0.63 ± 0.06 ^a^

Atomic ratios (%) are obtained from the low-resolution survey scan. Values represent means ± SDs. Different letters indicate significant differences (*p* < 0.05). S1, flower-bud stage; S2, pigmented stage; S3, unfold-petal stage; and S4, full-flowering stage.

**Table 2:** Analysis of C1s peaks of cell walls in *P. lactiflora* inflorescence stems.

Stage	Treatment	Element (%)				
		C1	C2	C3	C4	C5
S1	Control	28.19 ± 0.71 ^a^	43.83 ± 0.57 ^a^	17.71 ± 0.15 ^a^	8.31 ± 0.43 ^d^	1.96 ± 0.13 ^ab^
	Nano-CaCO_3_	28.70 ± 3.44 ^a^	44.05 ± 2.47 ^a^	15.70 ± 0.46 ^b^	9.67 ± 0.32 ^a^	1.87 ± 0.09 ^abc^
S2	Control	29.29 ± 3.02 ^a^	44.24 ± 0.38 ^a^	15.22 ± 0.62 ^bc^	9.00 ± 0.56 ^abc^	1.78 ± 0.22 ^bcd^
	Nano-CaCO_3_	30.78 ± 5.76 ^a^	43.93 ± 3.28 ^a^	14.53 ± 0.67 ^cd^	9.14 ± 0.53 ^ab^	1.61 ± 0.13 ^de^
S3	Control	26.99 ± 2.81 ^a^	44.02 ± 2.27 ^a^	17.92 ± 0.11 ^a^	8.97 ± 0.38 ^bcd^	2.09 ± 0.05 ^a^
	Nano-CaCO_3_	28.94 ± 1.66 ^a^	43.79 ± 1.31 ^a^	15.96 ± 0.55 ^b^	9.45 ± 0.26 ^ab^	1.86 ± 0.01 ^abc^
S4	Control	29.52 ± 1.46 ^a^	44.54 ± 1.08 ^a^	15.28 ± 0.75 ^bc^	8.92 ± 0.43 ^bcd^	1.71 ± 0.14 ^cd^
	Nano-CaCO_3_	31.80 ± 1.49 ^a^	45.12 ± 1.07 ^a^	13.64 ± 0.69 ^c^	8.44 ± 0.36 ^cd^	1.43 ± 0.11 ^e^

Quantification of the functional group composition was obtained from the high-resolution spectra. Values represent means ± SDs. Different letters indicate significant differences (*p* < 0.05). S1, flower-bud stage; S2, pigmented stage; S3, unfold-petal stage; and S4, full-flowering stage.

## Supplementary Materials

The following are available online at https://www.mdpi.com/2073-4409/8/2/102/s1, Table S1: Gene-specific primers used in qRT-PCR analysis, Table S2: Summary of sequencing reads after filtering in *P. lactiflora* inflorescence stems with the Control and nano-CaCO_3_ treatment, Table S3: Quality metrics of unigenes in *P. lactiflora* inflorescence stems with the Control and nano-CaCO_3_ treatment, Table S4: Top 20 KEGG pathways of DEGs and DEPs in the Control and nano-CaCO_3_ treatment, Table S5: The correlation of 24 DEGs and DEPs in the Control and nano-CaCO_3_ treatment, Table S6: 26 differential metabolites in *P. lactiflora* inflorescence stems with the Control and nano-CaCO_3_ treatment, Figure S1: DEPs and its KEGG enrichment analysis.
