# Correction: Zhao et al. Integration of Transcriptome, Proteome, and Metabolome Provides Insights into How Calcium Enhances the Mechanical Strength of Herbaceous Peony Inflorescence Stems. *Cells* 2019, *8*, 102

**DOI:** 10.3390/cells11131994

**Published:** 2022-06-22

**Authors:** Daqiu Zhao, Yuhan Tang, Xing Xia, Jing Sun, Jiasong Meng, Jiali Shang, Jun Tao

**Affiliations:** 1 Jiangsu Key Laboratory of Crop Genetics and Physiology, College of Horticulture and Plant Protection, Yangzhou University, Yangzhou 225009, China; dqzhao@yzu.edu.cn (D.Z.); DX120180105@yzu.edu.cn (Y.T.); M160620@163.com (X.X.); jingsun@yzu.edu.cn (J.S.); jsmeng@yzu.edu.cn (J.M.); 2 Institute of Flowers and Trees Industry, Yangzhou University-Rugao City, Rugao 226500, China; 3 Ottawa Research and Development Centre, Science and Technology Branch, Agriculture and Agri-Food Canada, Ottawa, ON K1A 0A1, Canada; jiali.shang@agr.gc.ca

The authors wish to make the following changes to their paper [1]. Due to the authors having made an error, the control group and the control (enlargement) group in S3 and the nano-CaCO_3_ group and the nano-CaCO_3_ (enlargement) group in S4 of Figure 7A (marked in red) need to be corrected. Figure 7 should be changed from: 
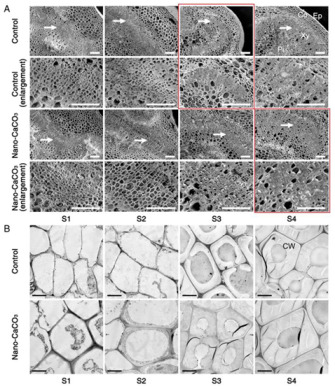


to: 
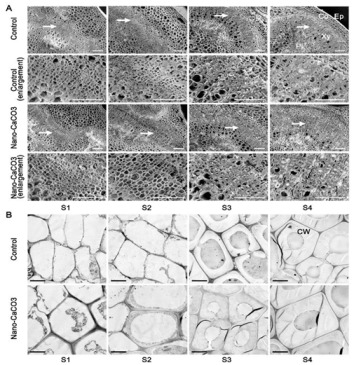


The authors would like to apologize for any inconvenience caused to the readers by these changes. The changes do not affect the scientific results. The original publication has also been updated.
